# Study on key influencing factors of competitive adsorption of coalbed methane by carbon dioxide displacement

**DOI:** 10.3389/fchem.2022.998592

**Published:** 2022-09-23

**Authors:** Xin Zhang, Gun Huang, Zhile Shu, Yao Tong

**Affiliations:** ^1^ School of Architecture and Civil Engineering, Xihua University, Chengdu, China; ^2^ State Key Laboratory of Coal Mine Disaster Dynamics and Control, School of Resources and Safety Engineering, Chongqing University, Chongqing, China; ^3^ School of Emergency, Xihua University, Chengdu, China; ^4^ Hoffmann Institute of Advanced Materials, Shenzhen Polytechnic, Shenzhen , China

**Keywords:** coalbed methane, competitive adsorption, different ash coal, influencing factors, CH_4_/CO_2_

## Abstract

The extraction of coal bed methane (CBM) by injecting CO_2_ into deeply buried unmined coal seams in competition with CH_4_ adsorption to provide a clean fuel is known as enhanced coal bed methane recovery (ECBM) and has proven to be an effective technological strategy to address global warming. The study of the interaction of coal with CO_2_ and CH_4_ under multi-physical field conditions is particularly necessary. In this work, a series of experiments were conducted on a home-made test system to investigate the competing sorption patterns of high and medium ash coal samples subjected to variables such as gas pressure, temperature, nodulation and lateral limit constraints. The results show that there is a sorption isotherm relationship between coal samples and exposure time. The adsorption capacity sorption of CH_4_/CO_2_ varied considerably for different ash coal samples. As the CO_2_ pressure increased from 2.3 to 5.5 MPa, the strain on the coal samples increased from 0.082 to 0.4%. The deformation in the vertical laminae direction is always greater than that in the parallel laminae direction. A correlation coefficient K exists between 1 and 2, and there is an internal expansion pattern in the adsorption deformation of coal. This paper can contribute to the improvement of ECBM efficiency.

## Introduction

In recent years, the demand for energy in China’s rapid economic development has gradually increased. At the same time, for the need for sustainable development, clean and efficient new energy sources such as coalbed methane and shale gas have been vigorously promoted. After mining for the past few decades, the shallow coal resources are increasingly exhausted, and most of the coal seam is buried deep underground. For some coal seams that do not have the mining conditions at the present technical equipment level, the method of extracting the clean energy gas coalbed methane (CBM) by injecting the carbon dioxide discharged from the industry to form competitive adsorption with the gas in the reservoir; It is called Enhanced Coal Bed Methane Recovery (ECBM). It has proved to be an effective strategy to mitigate global warming. However, the coal seam geology in China has a unique place, and there is a solid regional nature, so most areas show the characteristics of low pressure, low permeability, and low saturation characteristics. Therefore, in addition to the Qinshui Basin and the eastern margin of the Ordos Basin, it is not easy to achieve large-scale industrial development in other areas.

Many Scholars have carried out a lot of theoretical analysis and experimental research on coal permeability since the 1970s. According to the current research results, coal seam permeability is the main influencing parameter of coalbed methane extraction rate. Various factors will also affect it; these results are mainly concentrated in coal adsorption, gas pressure, effective stress, temperature and so on. Under certain conditions, Goodman, Larsen, Liu, and others found that the softening of coal seam with gas adsorption may be caused by the change of coal matrix gap structure in the adsorption process (Larsen, 2004; Goodman et al., 2005; Liu et al., 2010). Hol, Wang et al. believe that the permeability of adsorbed gases such as coalbed methane and carbon dioxide is affected by pore properties and the adsorption expansion effect (Hol et al., 2011; Wang et al., 2013). Palmer described in his study that the expansion and deformation of coal matrix induced by gas adsorption is a unique phenomenon of coal, which has a significant effect on pore fractures and will also affect coal seam permeability (Palmer, 2009). Many foreign scholars also come to the consensus that with the adsorption/desorption of gas in coal seams, coal seam expansion/shrinkage significantly impacts reservoir permeability (Harpalani and Chen, 1995; Seidle and Huitt, 1995; Chikatamarla et al., 2004; Kelemen et al., 2006; Cui et al., 2007; Bustin et al., 2008; Pone et al., 2010). Under constant pressure, Mazumder, Pan, Robertson, Wang, et al. found that gas permeability adsorbed by coal expansion decreased when pore pressure increased (Robertson and Christiansen, 2005; Mazumder and Wolf, 2008; Pan et al., 2010; Wang et al., 2010), Cui. Harpalani, Seidle et al. verified that when pore pressure decreases, coal shrinks and adsorbs gas permeability increases (Harpalani and Chen, 1995; Seidle and Huitt, 1995; Harpalani and Chen, 1997; Cui et al., 2007). Battistutta, Day, Levine et al. believe that coal’s expansion stress increases with pore pressure, and the expansion stress is reversible (Levine, 1996; Day et al., 2008; Battistutta et al., 2010). Palmer et al. proved that the permeability decrease is due to the increase of pore pressure and the reduction of effective stress under higher gas pressure (Palmer and Mansoori, 1996). Izadi et al. also confirmed that the decrease of permeability is the dominant factor of adsorption-induced strain at low pore pressure. The effectiveness decreases with the increase of pore pressure permeability rebound (Izadi et al., 2011). In the late seventies of the last century, Gawuga et al. studied the effect of stable aerodynamics between coal and gas seepage (Gawuga, 1979). Harpalani et al. studied the relationship between permeability and stress of gas-bearing coal samples under compression (Harpalani, 1985; Harpalani and Mcpherson, 1985). Somerton et al. found that the permeability of coal specimens decreases with the increase of pressure, so the empirical formula between permeability and stress (Somerton et al., 1975a) is established. At present, Durucan and Edwards have different effects on the permeability of varying coal samples, and the empirical relationship between permeability and stress of coal samples is obtained (Durucan and Edwards, 1986). Enever et al. found that by discussing the interaction mechanism between permeability and effective stress of gas-bearing coal and rock mass in Australian coal mines, There is an exponential relationship between the change of coal seam permeability and the transformation of *in-situ* stress. It is considered in the literature that the permeability in raw coal can be regarded as an application function of effective stress and pore pressure between fluids (Pomeroy and Robinson, 1967; Somerton et al., 1975b; Enever and Hennig, 1997; Pini et al., 2009; Siriwardane et al., 2009; Liu et al., 2011a; Liu et al., 2011b; Wang et al., 2011). Bae, Li et al. also concluded that permeability decreases with the increase in temperature (Bae and Bhatia, 2006; Li et al., 2010). Oldenburg believes that if the gas expands so that the temperature drops by more than 20°, the temperature will hurt permeability (Oldenburg, 2007). Long et al. injected N_2_, CH_4_ and CO_2_ into coal and conducted coal permeability experiments at different temperatures. It was found that the temperature change had a significant effect on the permeability, but the direct relationship between permeability and temperature could not be obtained (Long et al., 2009). Considering the effect of effective stress on fracture closure and pore pressure on matrix compression and promoting fracture expansion and adsorption deformation, EP Robertson and RL Christiansen established the stress-permeability model of pore fracture binary elastomer (Robertson, 2005; Robertson and Christiansen, 2006; Robertson and Christiansen, 2007a; Robertson and Christiansen, 2007b).

Dai analyzed the molecular dynamics method of methane and carbon dioxide’s absorption and diffusion properties at different burial depths and found the competitive adsorption relationship between methane and carbon dioxide (Dai et al., 2021). Li believes that the adsorption mechanism of CH_4_ and the competitive adsorption process between CH_4_ and CO_2_ still need to be explored in the coal seam at the microscope level, especially the water that hinders the adsorption isotherm of CH_4_, and the salinity reduces the adsorption capacity of CH_4_ (Li et al., 2021). I proposed that when the injection pressure is not higher than the initial reservoir pressure, the deformation of the coal matrix is mainly desorbed by CH_4_. When the injection pressure is higher than the initial reservoir pressure, the deformation is desorbed primarily by CH_4_, and the permeability increases in the early stage (Zhao et al., 2021). Wang, though Studying competitive adsorption characteristics of CO/CO_2_/CH_4_ multi-component low concentration gases in coal. Under the same pressure, the adsorption capacity of the desorption process is greater than that of the adsorption process. In the adsorption state composed of CO, CO_2_, and CH_4_, the gases restrict and influence each other, showing competitive adsorption behaviour (Wang et al., 2020). Zhou explored the effects of temperature and pressure on competitive adsorption and diffusion behaviour and concluded that the adsorption capacity of CO_2_/CH_4_ increased with stress but decreased with the rise in temperature (Zhou et al., 2019). Li uses simulation results to increase the total pore volume, porosity, and effective pore ratio of low-rank coal to high-rank coal, increasing its adsorption capacity. At the same time, the oxygen-containing functional groups on the pore surface of coal selectivity of CO_2_/CH_4_ decreased with the increase of coal grade (Li et al., 2019). By injecting N_2_ and CO_2_ into the coupled thermal-hydraulic-mechanical (THM) numerical model to enhance coalbed methane recovery (CBM), Fan verified the competitive adsorption of ternary gas in the THM field. The comprehensive action of air pressure and ground stress led to the evolution of reservoir permeability (Fan et al., 2020). Kang believes that the adsorption capacity and overall adsorption heat of coal decrease, and the reduction largely depends on the grade of coal (Kang et al., 2020). Liu found that the adsorption capacity of coal to methane is related to the content of vitrinite and inert body and the metamorphic grade of coal (Liu et al., 2019). Chong regards kerogen as the representative of organic matter. He finds that the cluster size distribution analysis means a significant degree of discontinuity in the micropores that adsorb carbon dioxide and methane.

In contrast, the micropores show continuity with adsorbed water (Chong et al., 2021). Dutka believes that the degree of coalification primarily affects decreasing adsorption capacity (about 89%), while the impact of the geothermal gradient is the second (Dutka, 2021). We found that the high surface roughness of pore structure is related to methane adsorption capacity. In contrast, mesopore and macropore volume and specific surface area positively correlate with ash yield and static content (Wei et al., 2019). Cao believes that the sedimentary environment increases the ash and mineral content of coal, fills the interlayer system, reduces the porosity, and reduces methane storage capacity (Cao et al., 2019). Wang and Menthe believe that ash yield affects pore structure and coal permeability (Mendhe et al., 2017; Wang et al., 2018). Mohanty observed a perfect correlation between the comprehensive effects of ash, moisture, and carbon on the adsorption capacity of the studied coal (Mohanty et al., 2018). Peng believes that high ash content dramatically reduces coal’s adsorption and seepage capacity (Peng et al., 2017). Chattaraj proposed that methane adsorption capacity was positively correlated with carbon content and vitrinite reflectance and negatively correlated with water, ash, and volatile matter (Chattaraj et al., 2019). Zhang and Ren also put forward a similar point of view: ash’s output affects adsorption performance (Ren et al., 2019; Zhang et al., 2020). Zeng studied the relationship between cleat, volume compressibility, and effective horizontal stress (Zeng and Wang, 2017). Niu has proved through a series of experiments that the permeability is anisotropic, manifesting in the permeability in parallel bedding plane direction is more significant than that in the vertical bedding plane direction (Beucher and Meyer, 1993; Niu et al., 2018; Niu et al., 2020).

From the above review of literature results on reservoir properties for coal, significant discrepancies have been identified. Laboratory measurement of these reservoir properties requires test coal samples, but some chose tectonic coal, and others used core samples. Experimental results of the tectonic coal body in the laboratory are larger than intact coal, contrary to the field results. Moreover, they always focused on the specific study of a particular factor. At the same time, confining pressure and temperature are hot topics and attract more people. Most people use the method of numerical simulation to discuss the possible relationship of competitive adsorption. However, few people have researched cleavage and constrained adsorption. To better understand the competitive adsorption relationship between CO_2_ and CH_4_ under different geological conditions, a comparative experiment was carried out in a self-made coal and gas adsorption-expansion deformation microscopic observation device. The competitive adsorption relationship of gas pressure, temperature, joint, constraint, and other variables to different ash coal was analyzed using the difference in adsorption-induced expansion.

## Experimental materials, system and method

### Experimental materials

**FIGURE 1:**
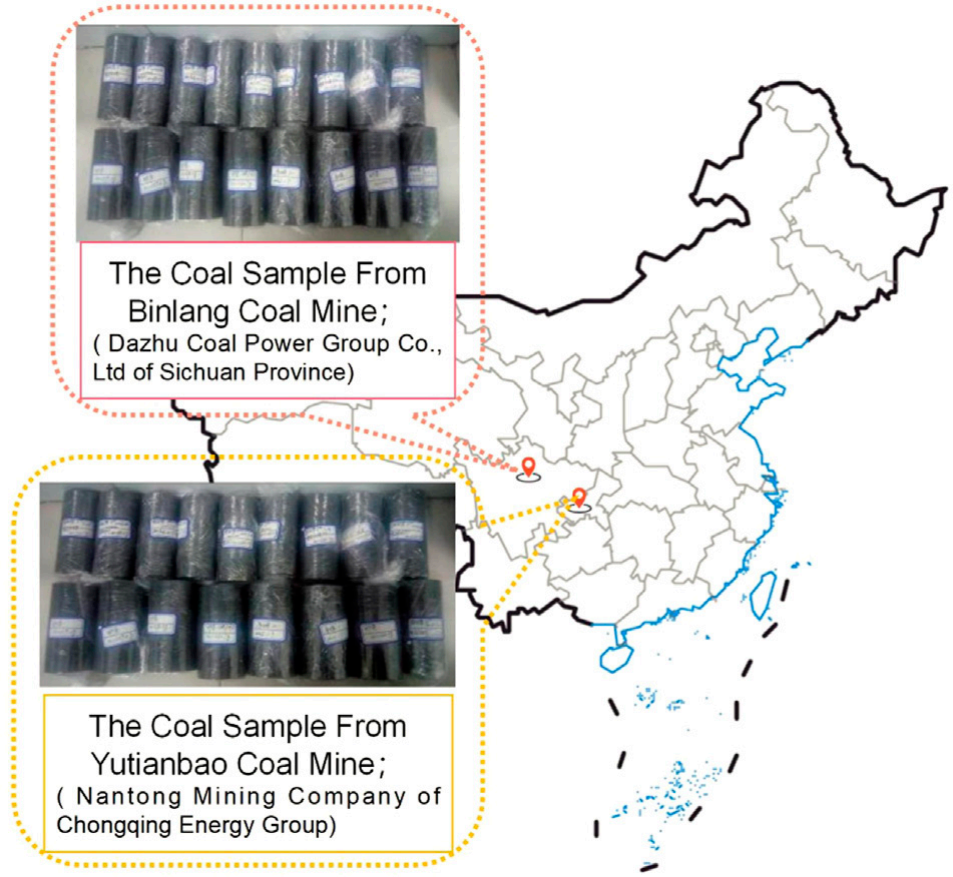
Experimental coal samples are collected and stored in the field.

The coal samples used in the experiments are core drill coal from the seam and will crush into raw. Which collected from the rom the Yutianbao Coal Mine (Nantong Mining Company of Chongqing Energy Group) and Binlang Coal Mine (Dazhu Coal Power Group Co., Ltd. of Sichuan Province),.China, as shown in Figure 1.

The coal components, including moisture (Mad), ash (Aad), volatile (Vad) and fixed carbon (Fc), were measured by infrared rapid coal quality analyzer. The results are shown in Table 1. According to the ash classification standard, the Yutianbao coal sample belongs to the medium ash coal sample, and the Binlang coal sample belongs to the high ash coal sample.

**TABLE 1:** Coal component test results.

Coal smaples	Moisture $\left(M_{ad}\%\right)$	Ash $\left(A_{d}\%\right)$	Volatile $\left(V_{d}\%\right)$	Fixed carbon $\left(FC_{d}\%\right)$
Yu’s	0.90	16.67	16.65	66.43
Bing’s	1.74	36.03	21.76	40.47

### Experimental system

**FIGURE 2:**
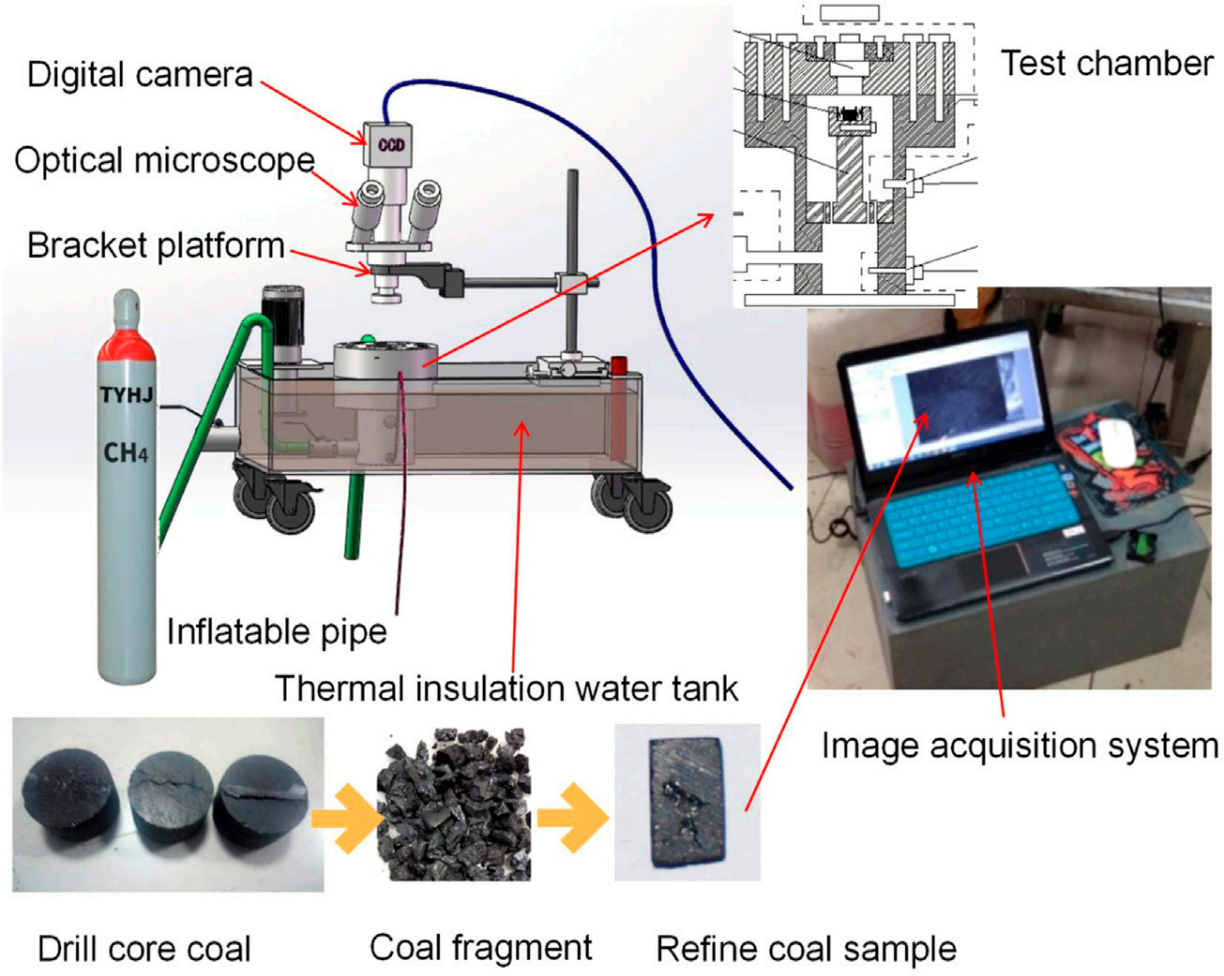
A new device for measuring adsorption-induced strain.

Unlike the traditional strain measuring device, we develop a piece of experimental equipment that can use accurate optical direct observation instead of the tedious strain gauge to obtain the time-varying adsorption expansion of coal, as shown in Figure 2, to realize the loading of stress at the end. The stress or displacement constraint adsorption experiment makes the test environment closer to the field situation. It has the advantages of a simple experimental procedure and a cheap test device. The specific description of the equipment is in the previous works of Zhang and Huang (Zhang and Huang, 2016).

### Experimental schemes

The coal samples (medium ash and high ash) taken from the previous site were broken and then processed into cuboid specimens of 3.5 mm*3.5 mm*7 mm to carry out experiments. The experiment was divided into two groups: no side limit and side limit. Each group of experiments were filled with CH_4_ and CO_2_ gas to carry out the adsorption experiment, and the input gas pressure was 2.3, 3.4, 4.1 and 5.5 MPa in turn, and the temperature in the test chamber was kept at (20°C, 30°C, 40°C) by heating the water tank with heating rod. In the course of the test, rely on the test system, continue to take photos to record the volume changes of coal samples.

### Information extraction technology of adsorption expansion based on digital image technology

**FIGURE 3:**
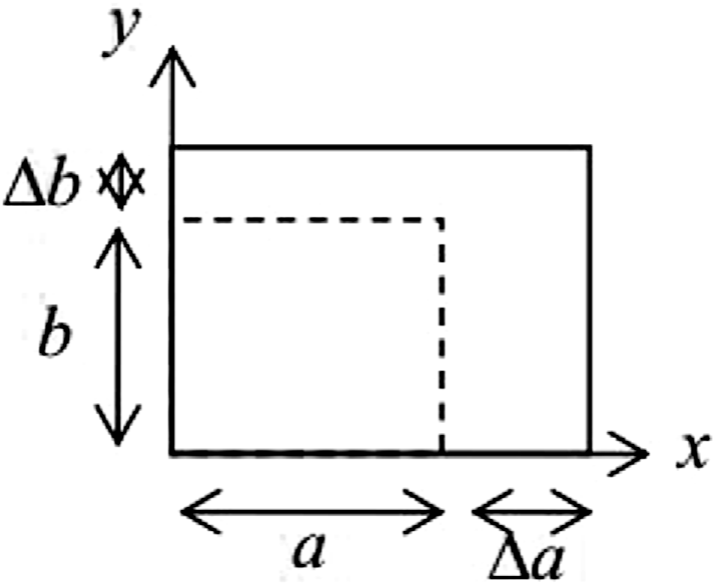
Deformation of a material element normal deformation.

From the knowledge of physics, we know that when a substance is subjected to a set of forces or is changing its states, such as a change in temperature, a change in water, or a chemical reaction, it changes size or shape. Will deform (see Figure 3), and the following equation can express its areal strain. Where *a* is the original length of the coal sample in the *x* direction, Δa is the length change, *b* is the original length of the coal sample in the *y* direction, Δb is the length change, A is the initial area, and ΔA is the area change.

$$\varepsilon_{areal}=\frac{\Delta A}{A}=\frac{\left(a+\Delta a\right)\left(b+\Delta b\right)-ab}{ab}=\frac{a\Delta b+b\Delta a+\Delta a\Delta b}{ab}=\varepsilon_{xx}+\varepsilon_{yy}+\varepsilon_{xx}\varepsilon_{yy}$$ (1)

**FIGURE 4:**
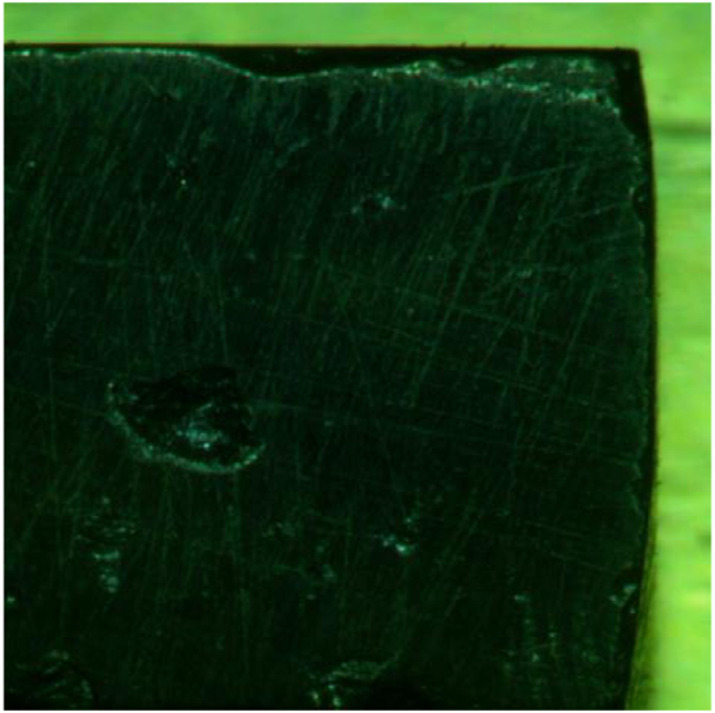
Real-Time State Image of Coal sample at a certain time.

**FIGURE 5:**
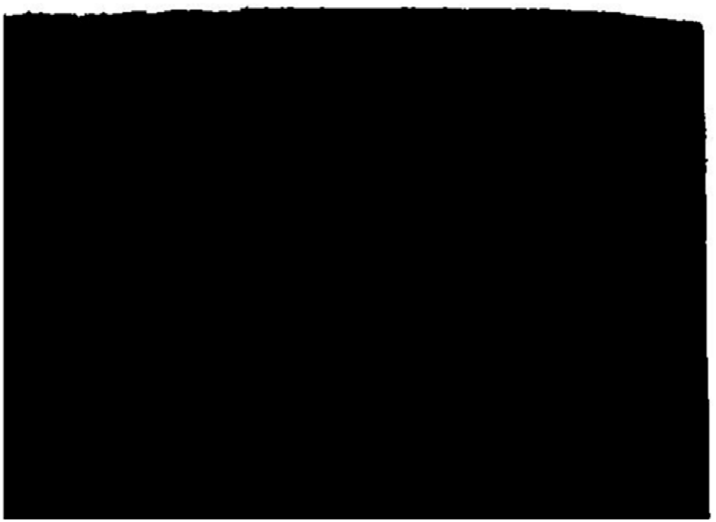
The result of watershed method for coal sample image.

The test sample is placed in the test device shown in Figure 1 with the help of a high-speed camera, we can arbitrarily obtain an image of a frame in the whole experiment, as shown in Figure 2. The obtained image was imported into Matlab program, and after binarization, the overall contour extraction of the whole coal sample is achieved by using the output result of the watershed algorithm (Xiong et al., 2020; Qi et al., 2021) (see Figure 4). We binarization each image just like Figure 5.

**FIGURE 6:**
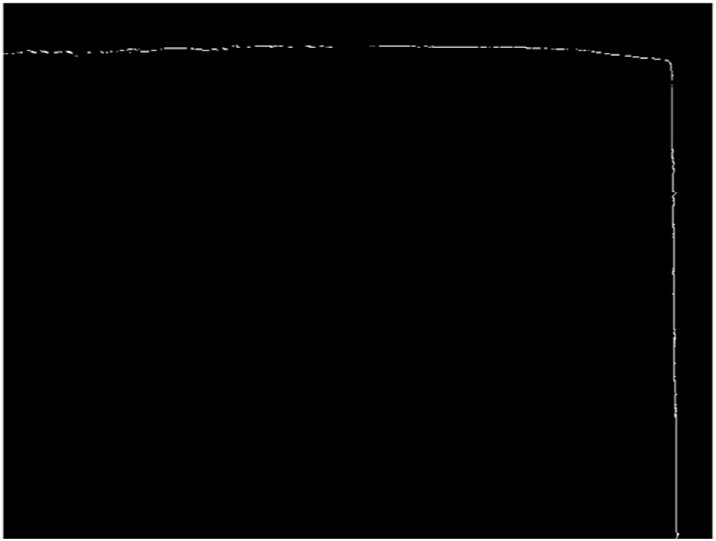
Image difference between *i* second and *j* second shows.

For a sequence of images, the difference between the two images can be obtained intuitively by comparing and calculating each pixel one by one. The difference between the two images is helpful for us to highlight the appearance difference and predict the trajectory of shape change (see Figure 6). We can describe this process with the following equation. Any image can be defined as a discrete two-dimensional array *f (x,y).* Each element in the matrix is called a pixel, where x and y are spatial coordinates. When the difference between the image value of the *i* second and the *j* second is outside the given threshold (*Tg*), we think that the point has shifted. And the point is defined as a white pixel point (a point equal to 1) by binary transformation (*d (x,y)*). Where 0 is a black pixel.

$$d_{ij}\left(x,y\right)=\left\{ \begin{array}{l} 1,\left|f\left(x,y,t_{i}\right)-f\left(x,y,t_{j}\right)\right|>T_{g}\\ 0,Other \end{array}\right.$$ (2)

**FIGURE 7:**
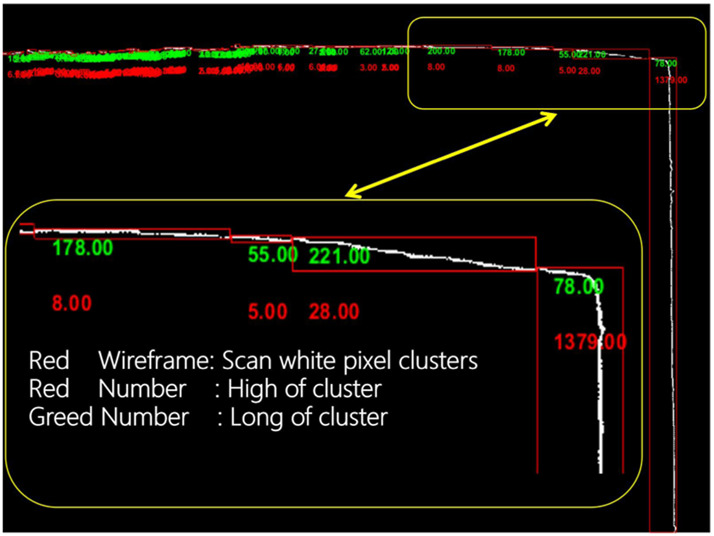
Feature extraction from differential images.

We calculate the area of each white pixel area in the output of the previous image difference comparison and then sum the area (see Figure 7), and the real-time strain data during the experiment can be obtained by Eq. 3. *P* is the Areal Strain, %.

$$P=\varepsilon {\left(M\right)}_{areal}=\frac{\sum areas_{i}}{Areas_{0}}$$ (3)

## Analysis of influencing factors of gas adsorption-expansion deformation of coal with different ash content

### Analysis of experimental results of the adsorption-induced strain of coal samples with different ash content

**FIGURE 8:**
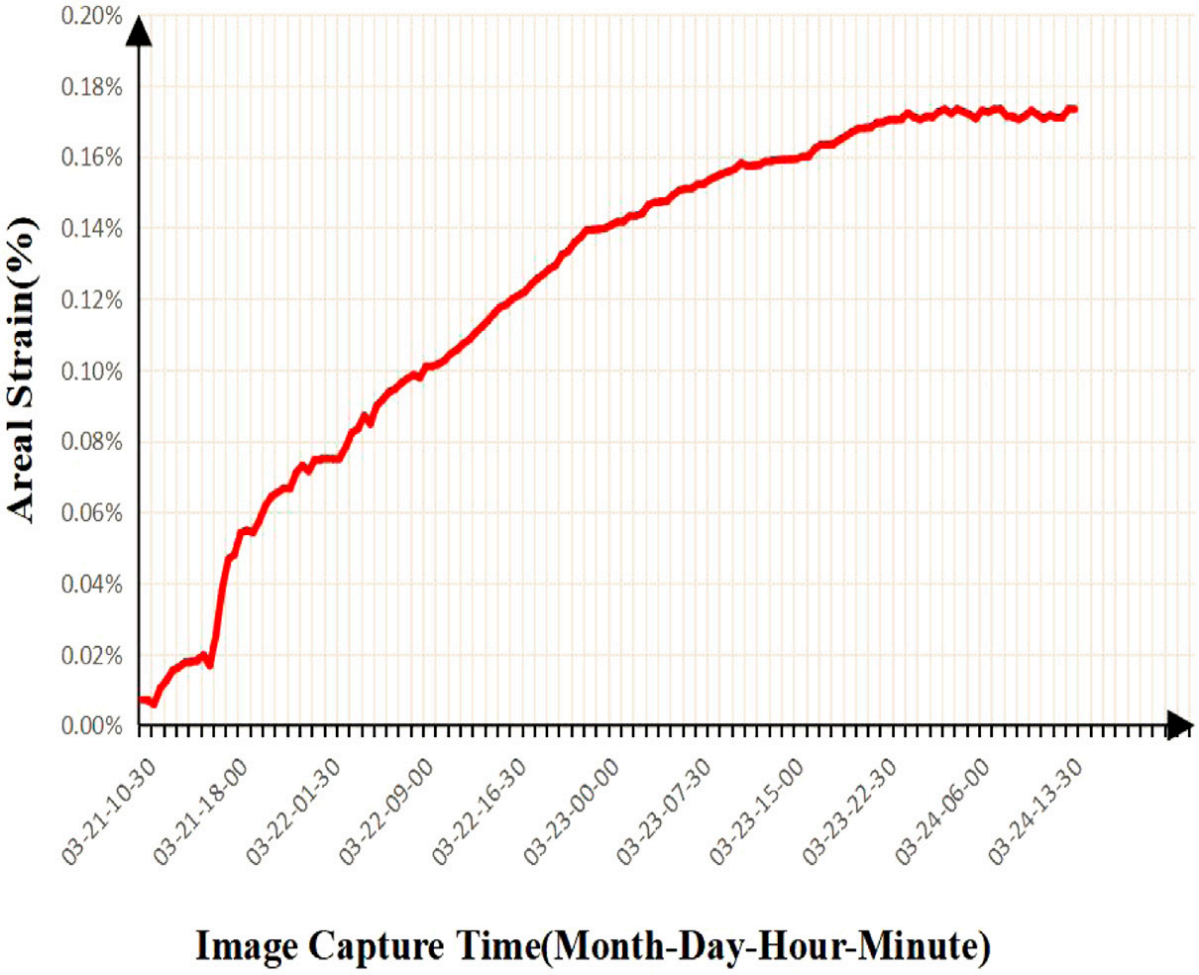
Results of time-dependent strain variation of ash coal filled with CH_4_ adsorption surface.

**FIGURE 9:**
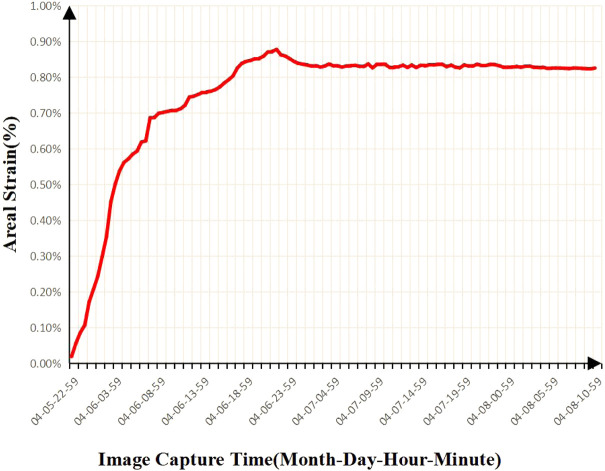
Results of time-dependent strain of ash coal filled with CO_2_ adsorption surface.

From Figure 8 and Figure 9, we can find that the adsorption strain has an adsorption isotherm relationship with the reaction time, and Robertson has seen a similar result in his work. He believes that the experimental results can be fitted by the Langmuir equation:

$$\varepsilon_{A}=\frac{\varepsilon_{A\max}T}{T_{L}+T}$$ (4)

Among them, $\varepsilon_{A}$ is the observed surface strain, also equal to $\frac{\Delta A}{A}$ in Eq. 1, $\varepsilon_{A\max}$ is the maximum strain-time constant, c represents the maximum strain produced by gas adsorption when the adsorption time is infinitely long at a given temperature and pressure. The time constant (T_L_) corresponds to the time required to reach half of the maximum strain value; T is the time at which the sample adsorbs under certain adsorption conditions (pressure, gas, temperature), which can be predicted that the observed strain ( $\varepsilon_{A}$ ) should be equal to the maximum strain ( $\varepsilon_{A\max}$ ) when the adsorption time is infinitely long.

The Lagergren quasi-first-order rate equation is based on the solid adsorption capacity. It can also be applied to the most common equation of adsorption kinetics of fluids. In the adsorption process, it is assumed that the rate ( $\frac{d\varepsilon_{A}}{dt}$ ) is proportional to the difference between the adsorption strain at time (t) and the adsorption expansion strain ( $\varepsilon_{A\max}-\varepsilon_{A}$ ). Defining k as a proportionality constant, we obtain the following equation

$$\frac{d\varepsilon_{A}}{dt}=k*\left(\varepsilon_{A\max}-\varepsilon_{A}\right)$$ (5)

Where $\varepsilon_{A}$ and $\varepsilon_{A\max}$ are the adsorption strain at a time and adsorption equilibrium, k is the first-order adsorption kinetic constant, assuming that when t = 0, $\varepsilon_{A}=0$ ; and when t = *t*, $\varepsilon_{A}=\varepsilon_{A\max}$ . After sorting out Eq. 4, we get the following equation:

$$\varepsilon_{A}=\varepsilon_{A\max}\left(1-e^{-kt}\right)$$ (6)

**FIGURE 10:**
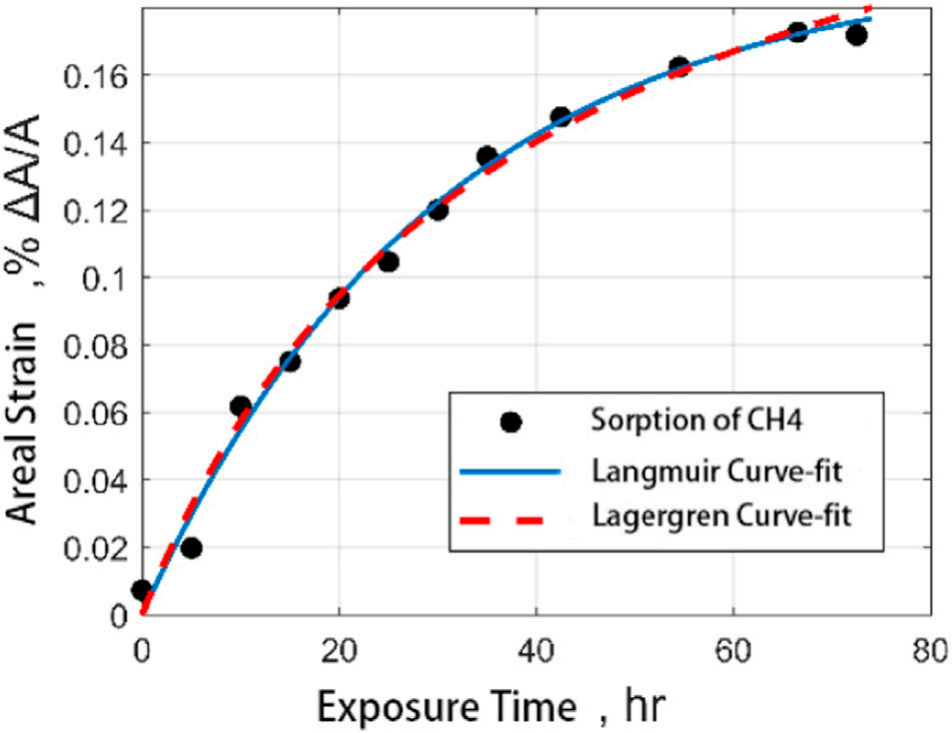
Comparison of fitting curves of medium ash coal filled with CH_4_ adsorption strain data with time.

**FIGURE 11:**
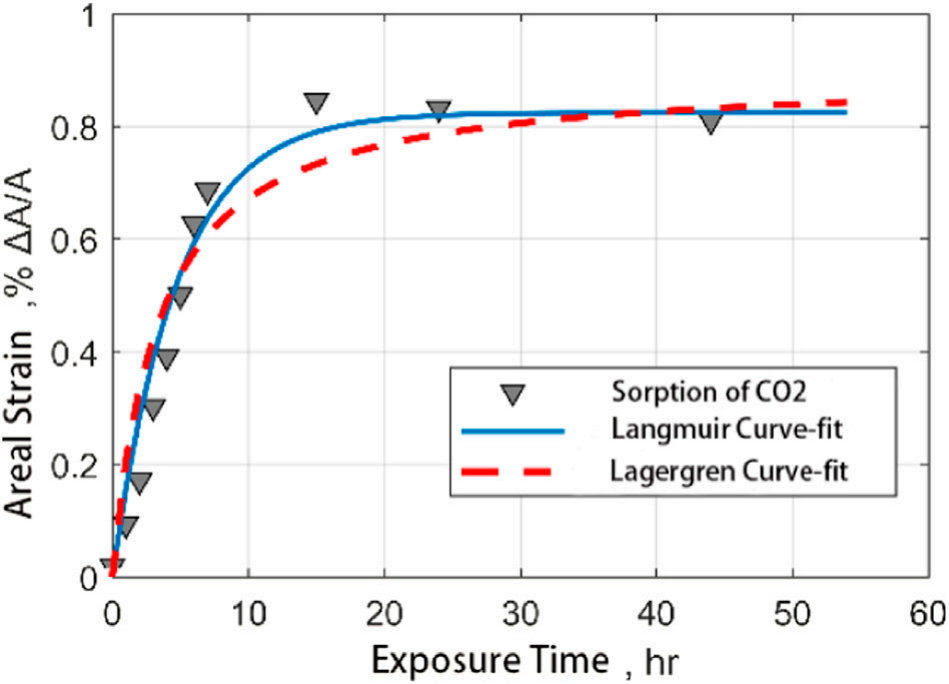
Comparison of fitting curves of ash coal filled with CO_2_ adsorption strain data with time.

The following figure shows the comparison between the fitting results of the Langmuir model and Lagergren model and the experimental results by using the output data of CO_2_ and CH_4_ adsorbed by coal samples. It is found that both models can well describe the process of gas adsorption by coal samples. However, the Lagergren model has a better correlation with the results of fitting the adsorption of CO_2_ on coal samples (Figure 10, Figure 11).

**FIGURE 12:**
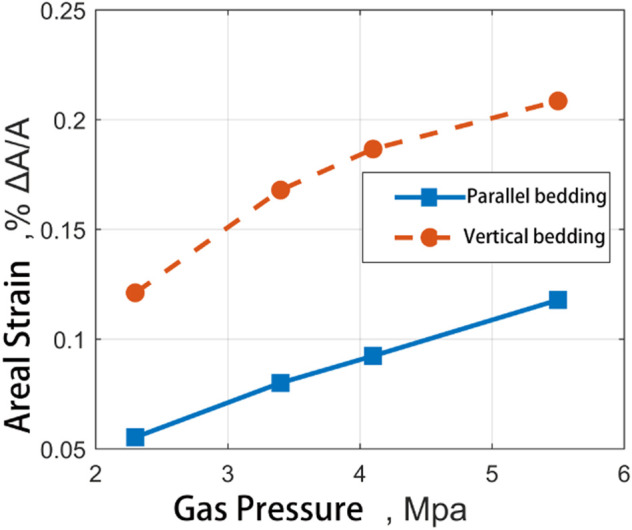
Comparative diagram of adsorption strain results of two kinds of coal samples in the direction of straight bedding and parallel direction of bedding with the change of input pressure of CH_4_.

Figure 12 shows the experimental results of adsorption-induced strain at different gas pressures of two groups of experimental materials. It can be seen from the figure that both the adsorption in CH_4_ and the adsorption in CO_2_, The adsorption strain junction of medium ash coal sample is always higher than that of high ash coal sample. There is no significant difference in strain results between the two at low pressure.

$$\left|\varepsilon_{Middle\;Ash\;Coal,2.3MPa,CO_{2}}-\varepsilon_{High\;Ash\;Coal,2.3MPa,CO_{2}}\right|=\left|0.823\%-0.741\%\right|=0.082\%,\left|\varepsilon_{Middle\;Ash\;Coal,2.3MPa,CH_{4}}-\varepsilon_{High\;Ash\;Coal,2.3MPa,CH_{4}}\right|=\left|0.171\%-0.152\%\right|=0.019\%$$

However, when the gas pressure reaches 5.5MPa, the adsorption-induced strain of CO_2_ in the medium ash coal sample is 1.411%, which was significantly higher than those of high ash coal samples under the same conditions (1.02%). At the same time, according to the trend of Langmuir curve fitting results in the figure, the difference will increase significantly with the increase in gas pressure.

Referring to the experimental results of particle size analysis of broken coal in Yutianbao Mine and Binlang Mine carried out by Luo (2016), Luo et al. (2016) it can be seen that there is little difference in the total pore volume between medium ash coal and high ash coal. The average values are 5.319mm3/g and 6.103mm3/g, respectively. However, the volume percentage of transition pores in medium ash coal samples is higher than that in micropores and mesoporous samples, accounting for about 70% of the total pore volume. Moreover, the surface area increases gradually with the decrease of particle size class. The surface area of coal particles with a particle size smaller than 0.075 mm is the most significant (Dai et al., 2021; Li et al., 2021). The results show that the adsorption capacity is also related to excessive pore volume, micropore volume, and specific surface area. The correlation coefficient is higher than excessive pore volume and specific surface area. The active specific surface area of adsorbed gas molecules increases, resulting in a significant increase in coal’s methanolic capacity, so coal’s adsorption capacity increases rapidly. At the same time, high ash content blocks some micropores, decreasing the active specific surface area of CH_4_ and CO_2_ gas adsorbed by coal, reducing Langmuir volume and decreasing the adsorption capacity.

**FIGURE 13:**
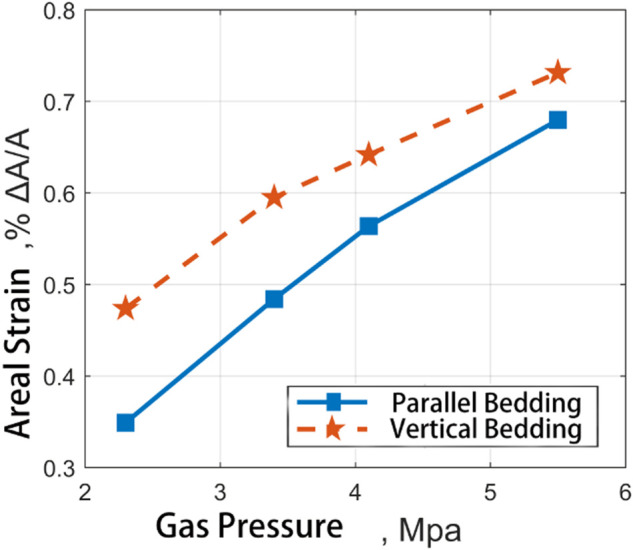
Comparative diagram of adsorption strain results of two kinds of coal samples in the direction of straight bedding and parallel direction of bedding with the change of input pressure of CO_2_.

With the help of the coal expansion information extraction program, the recognition, extraction, and statistics of dependent data in *x* and *y* directions can be realized. Taking the CH_4_ and CO_2_ adsorption-induced strain results of medium ash coal samples at different pressures (see Figures 12, 13), the strain results are counted according to *x* and *y* directions, respectively. According to the recording mark before coal sample processing, the vertical and parallel bedding directions correspond to each other.

It can be seen from the diagram that the results of adsorption CH_4_ induced strain of coal samples are very different in the vertical bedding direction and the parallel bedding direction. The adsorption-induced strain in the vertical bedding direction is more significant than that parallel to the bending direction. With the gas pressure increase, the adsorption-induced strain in the vertical bedding direction is more significant than that in the parallel. The ratio of the two remains in the range of 2:1. However, the vertical and horizontal ratio of CO_2_-induced strain on coal samples ranged from 1.35 at 2.3MPa to 1.08 at 5.5MPa, which was close to the increase of gas pressure. Therefore, the adsorption-induced strain in different directions is different for different gases.

Robyn et al. observed similar results in their work. The adsorption process is anisotropic, and the adsorption-induced expansion in the direction perpendicular to the layer surface is more massive than that parallel to the layer surface. Although the samples are entirely different, on average, the expansion in the vertical coal sample direction is 20–40% higher than that in the parallel coal sample direction (Fry et al., 2009; Zhao et al., 2021). Stuart et al.'s experimental data are only aimed at the adsorption-induced strain of coal in carbon dioxide. The anisotropic expansion model established by the model shows that when the pressure is greater than 6 MPa, The strain result parallel to the bending direction is about 60% (Day et al., 2008) of the strain perpendicular to the bending direction. Hol et al.'s data show that the elastic modulus is more significant in the direction parallel to the lamination plane (Hol et al., 2011). Saghafi et al. found that the diffusion rate parallel to the bedding is 70–90 per cent faster than that perpendicular to the litter (Saghafi et al., 2007; Wang et al., 2020).

The reason for the results of the appeal experiment is that in the process of the formation of cracks in the coal matrix, their direction is controlled by the tectonic stress, the plane joints are parallel to the maximum compressive stress, and the end joints are divided into groups of vertical and plane joints, which are discontinuous and rough. The anisotropic orientation distribution of the macerals (that is, their preferred orientation parallel to the bedding), coupled with the composition stratification of the material, leads to the anisotropic strength and various swelling characteristics so that the microcracks are parallel to the bedding. The expansion mechanism of coal is mainly the adsorption expansion of coal and the compression of gas pressure, and the compression effect of gas pressure on coal is independent of the type of gas. Therefore, regardless of the kind of gas, the three-dimensional expansion in space should be the same for a given amount of absorption. However, the same extension corresponds to a different expansion space, resulting in the observed behaviour.

Therefore, the surface strain can be characterized by the following equation:

$$\varepsilon_{A}=\frac{\Delta A}{A}=\frac{\left(l_{per}+\Delta l_{per}\right)\times \left(l_{par}+\Delta l_{par}\right)-l_{per}\times l_{par}}{l_{per}\times l_{par}}=\varepsilon_{per}+\varepsilon_{par}$$ (7)

Where $\varepsilon_{per,Area}=K_{gas\;with\;pressure}\varepsilon_{par,Area}$ , $K_{gas\;with\;pressure}$ is defined as the ratio of fracture plane joints to end joints, $K_{gas\;with\;pressure}\in \left[1,2\right]$

### Analysis of experimental results of adsorption-induced strain in different temperature environments

**FIGURE 14:**
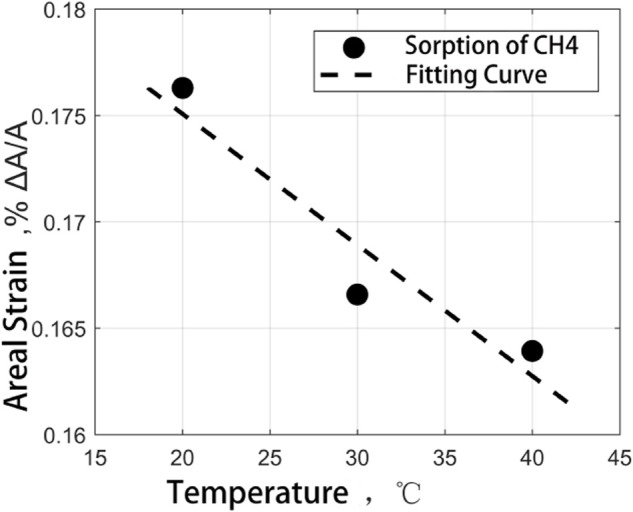
CH_4_ adsorption strain results when coal sample varies with experimental temperature.

**FIGURE 15:**
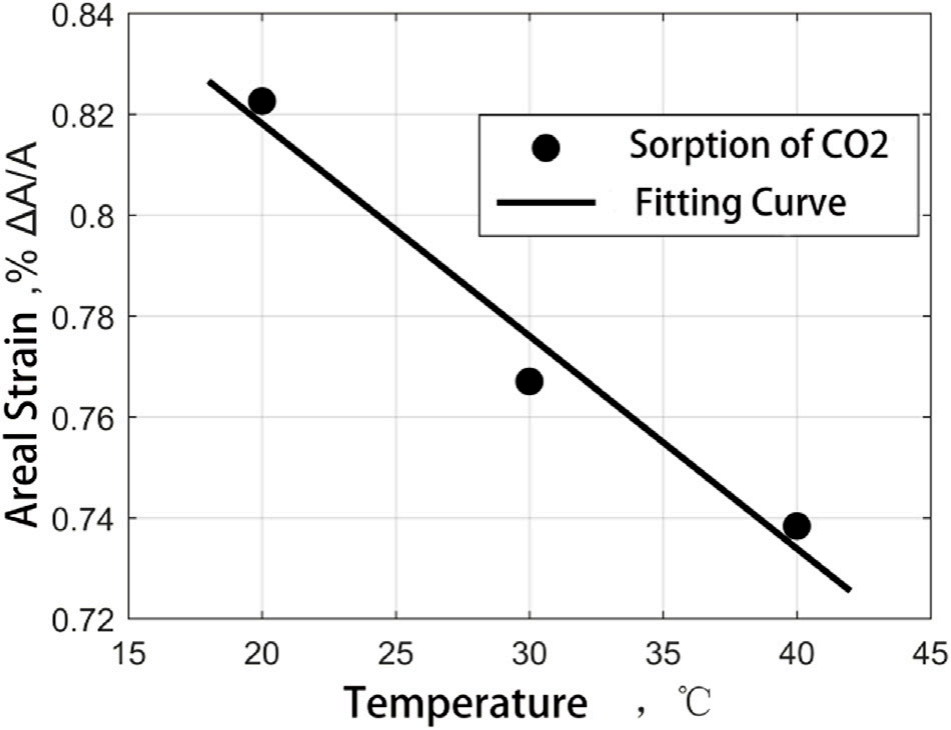
CO_2_ adsorption strain results when coal sample varies with experimental temperature.

To compare the effects of different temperature environments on the adsorption-induced strain experiments of coal samples, according to the previous experimental results, the laws of the two kinds of coal samples have been found to save time and experimental consumables. The adsorption-induced strain experiments of medium ash coal samples at different temperatures at 20°C, 30°C, and 40°C were carried out in CH_4_ and CO_2_ adsorption environments under 2.3 MPa gas pressure. The experimental results are shown in Figure 14 and Figure 15.

It can be seen from the diagram that the adsorption-induced strain of the coal sample is negatively correlated with the temperature. When the temperature increases from 20 to 40°C, The maximum strain of adsorption equilibrium of coal samples in CH_4_ and CO_2_ decreases slightly (0.012%, 0.084%, respectively), so the ratio of temperature to strain can be expressed as a linear function of temperature:

$$\frac{T}{\varepsilon_{T}}=f\left(T\right)=aT+b$$ (8)

Where T is the temperature, $\varepsilon_{T}$ is the strain value at T degrees Celsius; a, b is the constant related to the temperature function, their fitting results are shown in Table 2

**TABLE 2:** Strain-temperature fitting constants of coal samples for different adsorbed gases.

Gas type	Temperature relation constant	
	A	B
CH_4_	6.561	-17.592
CO_2_	1.49	-5.493

Although the strain value of coal samples decreases with the increase of temperature in the CH_4_ and CO_2_ gas environment, there are different downward trends, which can be characterized by constant a. The difference between the two constants is closely related to the boiling point of the gas, and it is generally believed that the gas with a high boiling point has more robust adsorption properties. In the figure, the adsorption-induced strain results of coal in CH_4_ and CO_2_ adsorption gases at different temperatures show a slight decrease with the increase in temperature. It can be regarded as the effect of partial adsorption gas desorption on the surface tension of the coal matrix in dynamic equilibrium and the competitive coupling result of thermal expansion and desorption shrinkage.

### Adsorption expansion experiment of coal under constrained conditions

The experiments in the previous section are all carried out under unconstrained conditions. According to Liu and Chen, many experimental results are compared with the field test results. The free expansion condition and the constraint condition in all directions represent the upper and lower bounds of the permeability evolution model. The field results are more consistent with the constant volume condition results, that is, the displacement constraint condition in all directions (Liu et al., 2011b; Chen, 2012; Zhou et al., 2019).

**FIGURE 16:**
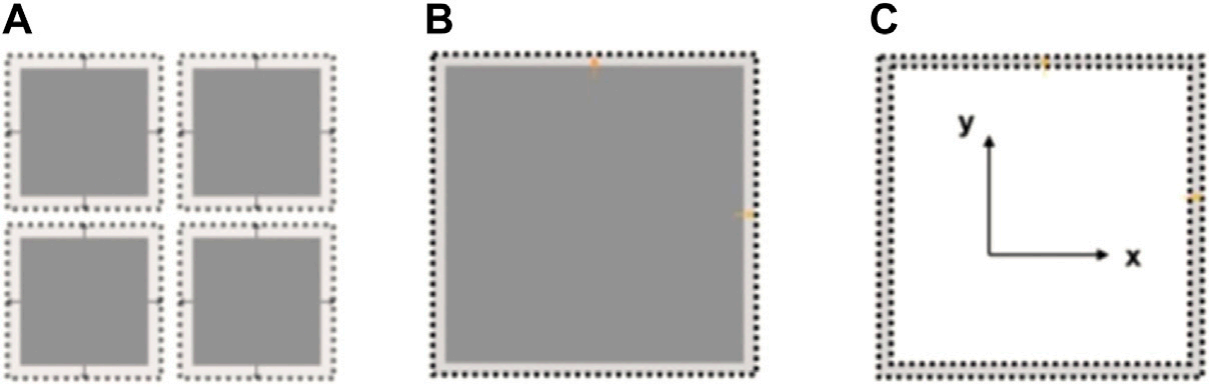
Schematic diagram of unconstrained adsorption induction experiment. **(A)** Matrix medium model with pores; **(B)** Continuum matrix model; **(C)** Matrix expansion.

Due to the limitation of test conditions, we can not complete the complete displacement constraint test. Therefore, we make the following assumption; if the loading mode is assumed to be two extreme cases, then it can be regarded as an unconstrained adsorption induction experiment compared with an adsorption induction experiment with displacement constraints, as shown in Figure 16.

As shown in Figure 16, it is assumed that the coal matrix frame in the coal sample can be seen in space as a separate block thoroughly segmented by penetrating fissures, with a horizontal section as shown in Figure 16A. For the unconstrained model, because the pore pressure is equal to the given ambient gas pressure in the adsorption equilibrium, the adsorption-induced expansion of the coal matrix will only lead to the development of the block. However, it will not change the space size of the cracks in it. Therefore, the adsorption-induced strain of the coal matrix at this time will not affect the permeability of the fissure. At the same time, the image result is output through the development of the coal gas adsorption-expansion deformation microscopic observation device appealed. The expansion of coal and rock that we have observed is shown in Figure 16B. It should be a steady expansion in three directions and processed by adsorption expansion information extraction technique. The experimental results are shown in Figure 16C.

**FIGURE 17:**
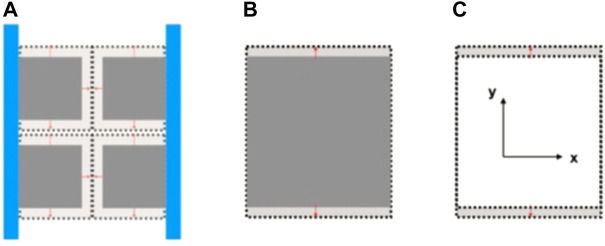
*X*-axis displacement-constrained adsorption induced strain experiment schematic diagram. **(A)** Matrix medium model with pores; **(B)** Continuum matrix model; **(C)** Matrix expansion.

However, when XYZ’s omnidirectional external displacement constrains the coal sample, we think the coal matrix is a pore elastomer. Then the same expansion will be wholly transformed into increasing the internal fracture space and squeezing the interior fracture space. We can call it an “internal expansion” phenomenon. For the fully constrained model, the expansion strain will promote the change of coal permeability. For the fully constrained model, the optical observation device cannot penetrate the metal and coal body to observe the real-time change of the internal fissure space due to the adsorption “internal expansion” process. Therefore, we only realize the displacement constraint in the *x*-direction through the metal block on the *x*-axis, as shown in Figure 17A. The stiffness of the metal limit block is much higher than that of the coal body. In the adsorption expansion under constrained conditions, the expansion amount which should belong to the *x*-direction will be wholly transformed into “internal expansion.” When the adsorption equilibrium is reached, This internal expansion will completely seal the interior fracture space while affecting the expansion in the *y*-direction and *z*-direction. At the same time, through the image output of the adsorption observation device under the multi-field coupling of the appealed coal and rock, the coal and rock we observed expand in the *y*-direction, as shown in Figure 17B, and at the same time, after processing by the adsorption expansion information extraction technique, The experimental results are shown in Figure 17C.

If a small part of the coal body relative to the whole volume is taken from the gas-bearing coal seam, they are divided into a solitary solid by stratification and termination in the space, and its adsorption process accords with the operation of free expansion. However, when the gas-bearing coal seam is analyzed from a macro point of view, it has complete transverse constraints and constant covering stress. Its reaction mechanism should be regarded as a collection of continuous volume models composed of many free expansion models.

**FIGURE 18:**
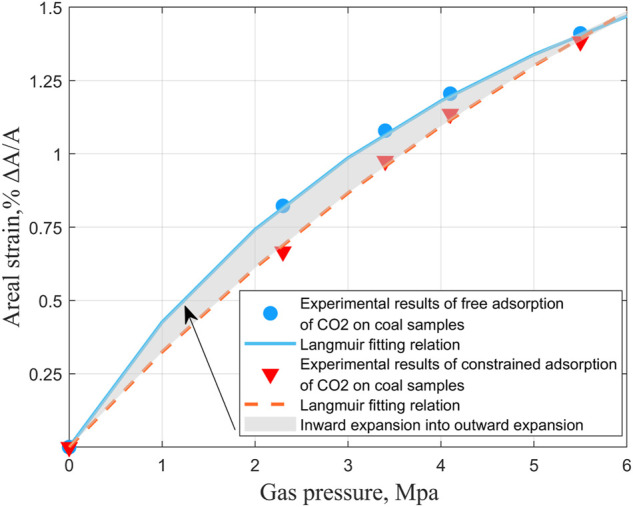
CO_2_ experimental results of free adsorption and constrained adsorption of coal samples under different gas pressures and comparison of fitting curves.

To compare the effects of two extreme loading conditions on the adsorption-induced strain experiment of coal samples. According to the previous experimental results, the other aspects of the two kinds of coal samples have been found. To save time and experimental consumables, we only conducted CO_2_ adsorption-induced strain experiments under different gas pressures at 20°C for medium ash coal samples. We compared them with the unconstrained test results under the same conditions. As shown in Figure 18.

It can be seen from Figure 18 that gas pressure plays a primary role in the adsorption and expansion of gas by coal, whether constrained or not. Combined with the analysis results of the influence of anisotropy in the previous section on the experimental results of the adsorption-induced strain of coal samples during the formation of cracks in the coal matrix, their direction is controlled by tectonic, and the plane joints are parallel to the maximum compressive stress. However, the end joints are vertical, and plane joints are in groups, most of which are discontinuous and rough. The anisotropic orientation distribution of the macerals (that is, their preferred orientation parallel to the bedding), coupled with the composition stratification of the material, leads to the anisotropic strength and various adsorption expansion characteristics so that the microcracks are parallel to the bedding. The expansion mechanism of coal is mainly the adsorption expansion of coal and the compression of gas pressure, and the compression effect of gas pressure on coal is independent of the type of gas. Similar studies have shown that even under unconstrained stress control conditions, the injection of adsorbed gas will reduce the permeability of coal under lower gas pressure. In comparison, the permeability of coal may rebound under higher gas pressure (Harpalani, 1985; Harpalani and Mcpherson, 1985; Seidle and Huitt, 1995; Harpalani and Chen, 1997; Siriwardane et al., 2009; Izadi et al., 2011). Therefore, regardless of the kind of gas, the three-dimensional expansion in space should be the same for a given amount of absorption. However, the same expansion corresponds to different expansion spaces. As a result, the upward expansion of the observation parties is not equal. If a displacement constraint is added to the axial direction based on free expansion, part of the outward expansion deformation that originally occurred in this direction will instead squeeze the fracture pore space into the coal matrix until it is closed, The excess energy is not completely dissipated, and the work is done along the direction of the remaining free surface. Under the condition of low pressure, the constrained expansion is less than the free expansion, the gas pressure is increased, and the deformation results are close to each other.

## Conclusion

The migration of coalbed methane in coal matrix is a process of solid-heat-flow coupling, that is, the complex process in which coal seam permeability depends on fracture pore pipe diameter, fluid pressure, temperature and loading force. The different induced strain characteristics of different gases adsorbed by coal matrix, the gas-filled with different pressures to produce the corresponding volume expansion, heating will generate the similar volume expansion and the corresponding thermal stress increment; loading mode will also be different. Different strain results, The loading mode is assumed to be two extreme cases, simplified as the private expansion behaviour under the constraints of free adsorption expansion and displacement. The main results are as follows:

1) A new test device and a suitable test method are adopted. Optical and non-contact measurement is an innovative testing method. An algorithm for automatic identification of adsorption expansion deformation of coal samples is developed using the Matlab program. There is an adsorption isotherm relationship between the adsorption strain and the reaction time, and the experimental data show that the method is feasible.
2) The adsorption capacity of coal samples with different ash content is different, and the adsorption difference of CH_4_/CO_2_ is also distinct. Under the condition of the 2.3 MPa test at room temperature and air pressure, the CO_2_ adsorption deformation of medium ash coal samples is 0.082% more than that of high ash coal samples, and the difference is only 0.0019%. This gap increases with gas pressure, and the deformation difference between the two coal samples adsorbing CO_2_ is as high as 0.4% under 5.5 MPa.
3) The anisotropy of the coal sample influences the result of adsorption deformation. The deformation in the vertical bedding direction is always more significant than that parallel to the bedding direction, and there is a relation coefficient K between one and 2. The result of adsorption deformation should be the sum of the deformation results in two directions.
4) Whether a displacement constraint on the coal sample adsorption expansion test influences the test results. This effect decreases with the increase of gas pressure, which proves an internal expansion mode of coal adsorption deformation.

## Data Availability

The original contributions presented in the study are included in the article/supplementary material, further inquiries can be directed to the corresponding authors.
